# Alternatives to Fight Vancomycin-Resistant *Staphylococci* and *Enterococci*

**DOI:** 10.3390/antibiotics10091116

**Published:** 2021-09-16

**Authors:** Benjamin Baëtz, Abdelhakim Boudrioua, Axel Hartke, Caroline Giraud

**Affiliations:** Normandie Univ., UNICAEN U2RM-Stress and Virulence, Esplanade de la paix, 14000 Caen, France; benjamin.baetz@unicaen.fr (B.B.); Abdelhakim.Boudrioua@med.uni-tuebingen.de (A.B.); axel.hartke@unicaen.fr (A.H.)

**Keywords:** Gram-positive, vancomycin, *Staphylococci*, *Enterococci*, antibiotics, antimicrobial peptides, bacteriophages, nanoparticles

## Abstract

Gram positive pathogens are a significant cause of healthcare-associated infections, with *Staphylococci* and *Enterococci* being the most prevalent ones. Vancomycin, a last resort glycopeptide, is used to fight these bacteria but the emergence of resistance against this drug leaves some patients with few therapeutic options. To counter this issue, new generations of antibiotics have been developed but resistance has already been reported. In this article, we review the strategies in place or in development to counter vancomycin-resistant pathogens. First, an overview of traditional antimicrobials already on the market or in the preclinical or clinical pipeline used individually or in combination is summarized. The second part focuses on the non-traditional antimicrobials, such as antimicrobial peptides, bacteriophages and nanoparticles. The conclusion is that there is hitherto no substitute equivalent to vancomycin. However, promising strategies based on drugs with multiple mechanisms of action and treatments based on bacteriophages possibly combined with conventional antibiotics are hoped to provide treatment options for vancomycin-resistant Gram-positive pathogens.

## 1. Introduction

The increasing spread of multidrug-resistant (MDR) bacteria is a global public health concern. Difficult-to-treat infections caused by MDR bacteria are responsible for an increase in morbidity, mortality, and healthcare costs [1,2,3]. Due to scientific and economic challenges, there are currently few novel drugs at the clinical stage with new mechanisms of action (MoA) [4]. The underlying economic issue regarding novel antibacterial drug development is mainly due to the early emergence of resistant strains that eventually limit new drugs’ lifespans and compromise the treatment of MDR bacterial infections [5]. In addition, although active drugs against antibiotic resistant priority pathogens listed by the WHO are urgently needed due to their acute virulence and multidrug resistance pattern [6], this same list based on anticipated patient need does not necessarily match the current market need. The global burden of MDR bacterial infections is predicted to increase in the coming years especially in locations where prevention and control measures of infectious diseases and antibiotic-use stewardship are not fully implemented [1,7].

The difficulty of identifying and developing new small molecules with broad spectrum antibiotic activity has helped to promote other strategies. Diverse drugs in terms of chemistry and MoA have recently emerged as potent alternatives to traditional approaches. Agents that are either not small molecules, do not act by directly targeting bacterial components necessary for bacterial growth, or both, are considered non-traditional approaches [7].

In this review, we focus on current available treatments against vancomycin resistant Gram-positive pathogens, promising drugs in preclinical and clinical stages, and finally, research prospects focusing on long term and sustainable solutions to tackle antibiotic resistance.

Vancomycin is a cell wall-active antibiotic approved in 1958 which was quickly reserved as a last-line antibiotic and has long been used to treat ampicillin resistant enterococci, methicillin resistant *Staphylococcus aureus* (MRSA), and *Clostridioides difficile* infections [8,9,10]. Despite supervised use, resistance to vancomycin appeared in the 1980s with the emergence of vancomycin resistant *Enterococci* (VRE) and *Staphylococcus aureus* (VRSA) driven by the acquisition of *van* resistance genes through horizontal gene transfer [11,12,13].

Vancomycin is a glycopeptide antibiotic targeting the peptidoglycan. The aglycone part of the compound forms hydrogen bonds with the dipeptide d-Alanyl-d-Alanine (d-Ala-d-Ala) of the *N*-acetylglucosamine-*N*-acetylmuramic acid-Pentapeptide of the peptidoglycan [14]. This drug-target complex blocks transglycosylation and transpeptidation steps of peptidoglycan biosynthesis and is the reason for its antimicrobial activity [15,16].

Acquisition of *van* genes clusters allows the synthesis of modified pentapeptide precursors. Different types of inducible vancomycin resistance operons have been described but VanA and VanB types are the most widespread and concerning from a clinical point of view [10]. Both clusters code for enzymes which leads to the modification of the dipeptide d-Ala-d-Ala to d-Ala-d-Lactate for which vancomycin has less binding affinity.

Expression of the *van* cluster is under control of the VanR/VanS two component system in VanA type resistance and VanR_B_/VanS_B_ in VanB type resistance. Autophosphorylation of the sensor kinase VanS occurs in the presence of both glycopeptides, vancomycin and teicoplanin, subsequently leading to the activation of the response regulator VanR which activates expression of *vanHAX* responsible for target modification. In contrast, VanS_B_ is activated only by vancomycin but not teicoplanin [13].

The continuous increase of vancomycin resistance in enterococci is of clinical concern. In 2014, vancomycin resistant *E. faecium* represented 10% of enterococcal clinical isolates in Europe. In 2019, vancomycin resistant *E. faecium* represented 18.3% of them (ECDC, EARS-Net, 2020) [17]. Compared to vancomycin susceptible isolates, the mortality of bacteremia caused by VRE is 2.5 times higher [18]. Therefore, efficient alternatives to vancomycin to treat VRE infections are urgently needed.

## 2. Alternatives to Vancomycin

### 2.1. Traditional Antimicrobials

#### 2.1.1. Conventional Antibiotics

**Figure 1:**
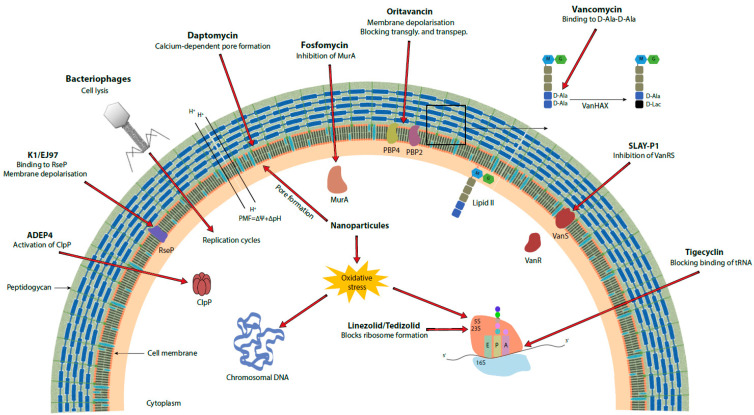
Mechanism of action of the different traditional and non-traditional strategies against vancomycin resistant Gram-positive pathogens. In bold, name of the molecule.

Several antibiotics have been tested or are recommended against microorganisms resistant to vancomycin and in particular VRE [19,20]. The first oxazolidinone recommended by the U.S. Food and Drug Administration (FDA) and then by the European Medicines Agency (EMA) was linezolid [20,21]. This bacteriostatic antibiotic binds to the 23S rRNA of the 50S ribosomal subunit thus preventing the formation of the 70S ribosomal complex and hence translation (Figure 1, Table 1) [22,23]. The G2576T mutation (from *Escherichia. coli* strain K12) in the gene encoding 23S RNA, as well as its duplication, allows resistance (MIC > 4 mg/L) to linezolid [20]. Some plasmids carrying genes for resistance to linezolid such as a methyltransferase encoded by *cfr*, which allows the methylation of the adenine residue 2503 of 23S RNA and thus resistance to five classes of antibiotics including linezolid, can spread between different bacterial species and genera [24]. Plasmids containing *cfr* are at risk of spreading due to their low fitness cost and the benefit they provide [20,25]. The *optrA* gene, also carried by a plasmid, codes for an ABC-F protein which also allows resistance to oxazolidinones and phenicol by a target protection mechanism [20,26,27]. The plasmid seems to be lost in the absence of selection pressure, which would limit its dissemination [28]. Similar resistance mechanisms are also found in *S. aureus* including MRSA strains [29]. Despite the presence of the above-mentioned resistance mechanisms, linezolid-resistant enterococci represent less than 1% of enterococcal infections in Europe, and the resistant strains are still sensitive to tigecycline and daptomycin [20,30]. On the other hand, several cases of enterococci resistant to both vancomycin and linezolid (LVRE) have been reported in recent years, which could ultimately prevent the use of this oxazolidinone against VRE [31,32]. These LVRE harbor the *van* operon and the G2576T mutation in most cases. Tedizolid may represent a viable alternative to linezolid in the treatment of skin and soft tissue infections [33]. The MIC of tedizolid is lower than that of linezolid in VRE and *S. aureus* [33]. Other antibiotics like nitrofurantoin or chloramphenicol have also been tested on urinary VRE isolates but were slightly less efficient than linezolid [34]. Quinupristin-dalfopristin have also been recommended for the treatment of VRE infections but later withdrawn because of the absence of bactericidal activity against *Enterococcus faecium* and their lack of activity against *Enterococcus faecalis* [35].

Daptomycin, a bactericidal antibiotic approved by the FDA and EMA for the treatment of skin and soft tissue infections as well as for *S. aureus* bacteremia, is a lipopeptide that acts by oligomerizing in the plasma membrane of Gram-positive bacteria in a calcium-dependent manner leading to pore formation, ion leakage, and death (Figure 1, Table 1) [22,37]. Several trials of treatments using daptomycin against VRE infections have been carried out, but these treatments appear to be associated with higher mortality than with linezolid in the case of bacteremia [38]. Nevertheless, this antibiotic is still active against VRE and seems to be preferable in certain types of infections such as endocarditis [35]. Non-susceptibility to daptomycin has already been reported, but these clinical isolates represent less than 1% of enterococcal infections worldwide [20,39]. For *S. aureus*, daptomycin resistance has also been reported, mostly based on an increase of the positive charge of the cell surface causing a repulsive effect of the antibiotic [40]. In addition, genes involved in cell wall synthesis and homeostasis allow low level vancomycin and daptomycin resistance in vancomycin intermediate *S. aureus* (VISA) [29,41]. In enterococci, several resistance mechanisms come into play, and they are different in *E. faecalis* and *E. faecium*. In *E. faecalis*, the three-component LiaFSR system, which participates in the regulation of envelope stress, appears to play an important role in adaptation to daptomycin. A deletion of Ile177 in LiaF (*liaF*∆ile177) triggers a change in the distribution of cardiolipin, which seems to influence the location of daptomycin binding away from the dividing septum of the bacteria [41]. It also appears that LiaR, the response regulator of the LiaFSR system, binds to other promoters allowing expression of *liaX*, *liaY*, and *liaZ* [41]. The functions of LiaY and LiaZ remain unknown, but LiaX has been characterized and its N-terminal and C-terminal domains appears to have distinct roles. The N-terminal domain binds to daptomycin in the extracellular environment, which leads to the activation of the envelope stress response via LiaFSR. The C-terminal domain inhibits the LiaFSR pathway and its truncation in daptomycin-resistant strains releases the inhibition, leading to cell-membrane remodeling [42]. Some mutations in *cls* coding for cardiolipin synthase, and *gdpD* coding for glycerol-phosphodiester phosphodiesterase, increase resistance of *E. faecalis* to daptomycin. The *gdpD* mutation has an effect on daptomycin resistance only if the *liaF*∆ile177 mutation is present. Resistance to daptomycin in *E. faecium* is mainly due to antibiotic repellence and can be acquired if *liaS* and *liaR* each have a specific mutation (T120A for LiaS and W73C for LiaR) [41]. Certain mutations in *yycFG*, a two-component system involved in cell homeostasis [43], may increase resistance to daptomycin, but the underlying mechanism at the basis of resistance is still unknown. Mutations in *cls* also appear to play a role in daptomycin resistance in *E. faecium* [40].

Tigecycline is a bacteriostatic antibiotic of the glycylcycline class indicated for the treatment of skin and soft tissue infections and intra-abdominal infections. It acts on both Gram-positive and Gram-negative bacteria by preventing the binding of the aminoacyl-tRNA/EF-Tu-GTP complex to the 30S subunit of the ribosome, which inhibits protein synthesis (Figure 1, Table 1) [20,104]. Although most resistances (MIC > 0.5 mg/L) are reported for Gram-negative bacteria, enterococci have also developed resistance mechanisms to this antibiotic. The clinical strains already reported have either the *tet(M)*, *tet(L)*, or both gene(s), which encode ribosome protective proteins (RPPs) that confers tetracycline resistance [20]. According to Dönhöfer et al. [44], the MoA of Tet(M) would be to sterically block the binding site of the antibiotic on the ribosome. Mutations in *rpsJ*, which encodes ribosomal protein S10, also lead to resistance in enterococci [20,45]. Although these tigecycline-resistant enterococci are still rare in the clinic (<1%) [20], a recent article reported a cluster of enterococci resistant to both tigecycline and vancomycin in a hospital in northern Germany [46]. Tigecycline-resistant MRSA have also been reported in Malaysia [47].

#### 2.1.2. Modified Antibiotics

One possible alternative could be the optimization of existing antibiotics to overcome bacterial defenses. Regarding VRE, the majority of research in this area focuses on the modification of glycopeptides.

Among these, the dimers of vancomycin were studied very early. The MoA of this antibiotic is thought to be related to the inhibition of the transglycosylase activity of penicillin binding protein 2 (PBP2) rather than by binding to the d-Ala-d-Lac precursors of monomers [50,51,52]. The advantage of this different MoA is inhibition of another step-in peptidoglycan synthesis and therefore restoring the sensitivity of VRE. Despite numerous studies on this dimeric vancomycin, to our knowledge there seems hitherto to be no clinical study for this new drug.

Another solution consists of modifying certain sites of the glycopeptides in order to improve their effectiveness or to extend their spectrum which can sometimes allow the molecule to acquire a new MoA [105,106,107]. This is, for example, the case for teicoplanin derivatives which show activity on both VRE VanB and VanA while teicoplanin has activity only on VanB strains [54]. The most promising strategy in the development of next-generation glycopeptides would be to modify the binding pocket of lipid II so that the molecule can bind both the d-ala-d-ala as well as the d-ala-d-lac version of the precursor [108,109].

There are currently glycopeptide derivatives that have been approved by the various regulatory agencies, which are telavancin, dalbavancin (formerly BI 397 [55]), and oritavancin (formerly LY333328 [55]). These lipoglycopeptides are indicated to treat complicated skin and soft tissue infections and telavancin has also been approved for the treatment of patients with ventilator-associated pneumonia but has subsequently been withdrawn from the European market [110]. Dalbavancin and oritavancin have activity against VRE and *S. aureus*, including against MRSA and VISA [56]. While dalbavancin has no activity on VanA strains, oritavancin has activity on both VanA and VanB strains [57,59,60]. It also exhibits in vitro activity on *S. aureus* biofilms [56]. The advantage of oritavancin over the other two molecules is that it has three distinct MoAs, namely, depolarization of the membrane, inhibition of transglycosylation, and inhibition of transpeptidation, which limits the emergence of resistance (Figure 1) [59]. Resistance has been demonstrated in the laboratory but not in the clinic [56,58].

In order to avoid this potential resistance development, new glycopeptides combining several MoAs in the same molecule would be promising [61]. For example, a vancomycin analogue with a modification in the lipid II binding pocket combined with two peripheral modifications, one of which is also present in oritavancin binds both the d-ala-d-ala end and the d-ala-d-lac end of lipid II, inhibits transglycosylation, and permeabilizes the bacterial membrane. This vancomycin analogue has a very low MIC, and, due to its triple action, showed limited risk of resistance development. This strategy of combining the MoAs, especially if they act synergistically, seems to be the key to obtaining antibiotics with long-lasting antimicrobial activity. This would provide a viable alternative over time to vancomycin in the case of VRE, but this strategy should be adaptable to other antibiotics as well. 

Finally, certain modifications on other classes of antibiotics such as aminoglycosides make it possible to obtain activity on VRE [111]. However, there are only few examples concerning the modification of previously ineffective antibiotic classes into effective drugs against enterococci.

The modification of antibiotics is a promising alternative in the fight against VRE provided that antibiotics with several MoAs are developed in order to limit rapid appearance of resistance in these multidrug-resistant bacteria.

#### 2.1.3. Combinations of Antibiotics

Using multiple antibiotics simultaneously is another strategy used in the clinical settings. Combining the advantages of different molecules can give rise to synergistic effects that make it possible to overcome certain resistances and limit their development and spread. This strategy is recommended in cases of infective endocarditis due to enterococci and other Gram-positive bacteria resistant to vancomycin [22]. 

There are only few examples of combinatorial treatments with vancomycin. In one of these studies the combination with daptomycin was tested, which had a synergistic bactericidal effect [62]. A combination of vancomycin with citrinin, a polyketide, reduced the MIC of vancomycin [63].

Linezolid has also been tested with many antibiotics. While rifampicin has an antagonistic effect in some studies [64], several antibiotics such as ceftriaxone, doxycycline, or fosfomycin act in synergy or have additive effects on bacteria resistant to vancomycin, at least in vitro [65]. Combinations of vancomycin with oritavancin have also been tested but have not shown better results than individual treatments with the antibiotics [66]. The combination of linezolid with fosfomycin is promising, as it has been shown that synergy is possible between these two molecules in the *G. mellonella* larvae model [67]. The authors supposed that fosfomycin, by acting on cell wall synthesis via MurA inhibition, promotes linezolid penetration, showing increased activity on both VRE [67] and MRSA (Figure 1) [68,69]. In addition, an activity on biofilms of vancomycin resistant *E. faecium* has been demonstrated with this combination [69].

Daptomycin is also the subject of many studies. As with the linezolid combination, rifampicin or oritavancin combined to daptomycin had either antagonistic activity or no synergistic effect, respectively [64,66]. Other antibiotics, especially β-lactams (ceftaroline, ertapenem, ampicillin, ceftriaxone, cefepime, and ceftobiprole) act in synergy with daptomycin to re-sensitize some daptomycin resistant VRE strains [39,70]. A synergistic effect is also observed between daptomycin and gentamycin or fosfomycin, and the latter combination is recommended by some authors for the treatment of VRE [22,64].

Tigecycline is recommended in combination with gentamycin but only as a last resort because of its limited clinical efficacy and its adverse effect profile [22]. Significant activity of tigecycline on biofilms of vancomycin resistant *E. faecalis* and MRSA when combined with Fosfomycin has also been reported [68,69].

Oritavancin is also being studied in combination with other antibiotics, but like combinations with linezolid or daptomycin, little benefit has hitherto been demonstrated. Smith et al. [71] thus found that there is no gain in activity in the presence of β-lactams in VRE and Meyer et al. [66] showed that oritavancin alone was as effective as in combination with ceftriaxone, gentamicin, or rifampicin to treat VRE in the *G. mellonella* model. One study reported synergy between oritavancin with gentamycin but only on strains sensitive to gentamycin and was therefore not recommended in the treatment of VRE [72].

Other antibiotic combinations are being tested to counter bacteria resistant to vancomycin, such as quinupristin-dalfopristin in combination with doxycycline which was predicted to be synergistic, although this has not been clearly proven yet [22].

Fosfomycin, already presented in combination with linezolid, daptomycin, and tigecycline, is one of the antibiotics that improve activity against bacteria resistant to vancomycin. This antibiotic also acts in synergy with teicoplanin or rifampicin with strong activity not only on VRE but also on MRSA [68,69]. On the other hand, the authors do not recommend combining it with ampicillin due to absence of synergy.

A very potent class of antibiotics are activators of the Clp protease ClpP subunit. ADEP4 is one of them and has high activity on Gram-positive bacteria. This antibiotic induces the autodigestion of cells by activation of ClpP and allows elimination of the pathogen even in the presence of high cell density (Figure 1) [73]. The problem with this antibiotic is that its target is accessory in bacteria, so using it in combination with other drugs is essential to avoid the fast appearance of resistance. In general, glycopeptides have antagonistic activity with this antibiotic [73,74]. All the antibiotics tested, namely, linezolid, ampicillin, ciprofloxacin, daptomycin, rifampicin, and tigecycline allow a marked decrease in the bacterial load in vitro, while limiting the development of resistance [73]. Oritavancin has an antagonistic effect in combination with ADEP4. Ampicillin appears to be the most effective antibiotic in combination with ADEP4 to treat VRE [73].

The use of several antibiotics simultaneously reduces the risk of treatment failures and the appearance of resistances in pathogens, but this strategy also has drawbacks. The antibiotic combinations used lack generally specificity and have a major impact on the microbiota. In addition, the majority of these data come from in vitro studies, and may differ from treatments of real-world infections in the clinic.

### 2.2. Non-Traditional Antimicrobials

#### 2.2.1. Antimicrobial Peptides and Bacteriocins

##### Antimicrobial Peptides

Antimicrobial peptides (AMPs) are host defense peptides found in both eukaryotes and prokaryotes. This part will focus on eukaryotic AMPs. They are part of the innate immune system in humans and have several activities including bactericidal activity [112]. 

AMPs have a variety of different MoAs. Some cause depolarization of the membranes by pore formation, and others modify intracellular processes such as transcription and translation, degradation of the cell wall or modification of the composition of the membrane [37,112].

There are several strategies for using peptides against vancomycin resistance. These can, for example, be designed to prevent the emergence of resistance. Mull et al. [75] synthesized a vancomycin antagonist peptide that binds covalently to vancomycin. This peptide is capable of post-treatment lowering of the concentration of the antibiotic in the colon, and therefore decreases selection pressure and hence resistance development.

Many antimicrobial peptides currently under study are cationic peptides [113]. These peptides integrate into the negatively charged bacterial membrane with their hydrophobic regions. This MoA is fast and the risk of resistance development of these peptides is generally low [112]. Furthermore, numerous peptides active against VSE and VRE have been reported [76]. 

Another approach is to use the antimicrobial peptides in combination with conventional antibiotics to treat infections with Gram-positive pathogens. Thus, many peptides allow re-sensitization of resistant strains to vancomycin. The SLAY-P1 peptide, for example, has a synergistic effect with vancomycin on VRE. The peptide inhibits the transcription of *van* genes under vancomycin exposure. The exact mechanism is unknown but the authors suggested that the peptide blocks the VanS sensor (Figure 1, Table 1) [76]. An antimicrobial cationic peptide derivative based on chitosan and lysine residues, called CSM5-K5, allowed re-sensitization of several pathogens against antibiotics for which they are resistant by perturbation of the bacterial membrane [77]. This peptide thus allows the re-sensitization of VRE to vancomycin and of MRSA to oxacillin [77]. It also helps reduce biofilm formation in combination with these antibiotics [77]. Some derivatives of the human antimicrobial peptide LL-37, namely, LL-13 and LL-17, demonstrated activity on VISA and a reduction in vancomycin MIC on VRSA in combination with vancomycin and also demonstrated anti-biofilm activity on these pathogens [78]. Umstätter et al. [79] fused vancomycin with a cationic peptide and this conjugate showed greater efficacy on VanA, VanB, and VanC strains than the glycopeptide itself. This derivative is less concentrated in the kidneys than vancomycin and its MoA is not linked to binding the D-ala-D-ala ends of pentapeptides [79].

The use of antimicrobial peptides is still limited due to their high production costs and their sensitivity to proteolytic enzymes limiting their in-host stability. Some peptides also have hemolytic activity, which seems to be correlated with increased positive charges of the peptides [112]. Although resistance development to these compounds is rare, some bacteria have nevertheless developed an arsenal of defenses. For example, bacteria produce proteolytic enzymes in the presence of antimicrobial peptides [112]. The modification of surface charges and the trapping or active efflux of peptides are other mechanisms of bacteria to withstand their toxic activities [114]. A strategy to avoid instability might be by sending the peptides directly to their targets with the help of nanocarriers that will protect the peptides until their delivery [112].

##### Bacteriocins

Bacteriocins are special types of AMPs ribosomally synthesized by bacteria, often targeting organisms phylogenetically close to the producing strain. Bacteriocin-producing strains have a selective advantage in their ecological niches by inhibiting the growth of competitors, and are produced by both Gram-positive and Gram-negative bacteria [19].

Bacteriocins are divided into three classes: class I contains bacteriocins with modified residues, called lanthipeptides or lantibiotics; class II, comprising small heat-stable non-modified molecules and which are subdivided into four categories; and class III, which are large and heat labile [115]. Members of the three classes have been used in the food industry for over 50 years against foodborne pathogens, but their use in the medical field is hitherto very limited [19]. Bacteriocins have different MoA that are similar to the antimicrobial peptides except that the membrane-targeting bacteriocins interact with proteins rather than purely physical interactions [19,81,86].

Several strategies have been tested to prevent or treat vancomycin resistant pathogen infections by bacteriocins. One example is the introduction of a consortium of bacteria by fecal transplantation to combat the presence of VRE in the colon of patients at risk [80]. One of the added strains, *Blautia producta* (BP_SCSK_), secretes a lantibiotic that reduces the density of enterococci in the colon of infected mice, and could therefore decrease the risk of VRE infections in critically ill patients. Other lantibiotics demonstrate efficacy against Gram-positive pathogens such as mersacidin or epidermin and gallidermin which have an effect on MRSA [81]. Lacticin 3147, representative of the two-peptides lantibiotics, binds to lipid II and integrates into the bacterial membrane resulting in the formation of pores. This lantibiotic not only has activity on MRSA, but also on VRE and other bacteria of clinical interest [83]. Another two-component lantibiotic, roseocin, also has an effect on MRSA and VRE, but lacks a lipid II binding motif. The MoA of roseocin is still unknown [116].

The MoA of the other classes of bacteriocins are also very diverse. For example, pumilicin 4, which is active against MRSA and VRE, seems to destabilize the bacterial membrane [84]. The K1 and EJ97 class II bacteriocins target the membrane-bound protease RseP, which is upregulated at the start of an infection. The spectrum of action of these molecules is restricted to *E. faecium* and *E. faecalis* (VSE and VRE), respectively [19,85]. The binding of K1 and EJ97 bacteriocins to their receptor RseP leads to the formation of pores which ultimately causes a depolarization of the membrane (Figure 1, Table 1). Although RseP is an accessory, bacteria not expressing this protease will be more sensitive to stress since RseP is needed for the release of an alternative RNA polymerase sigma factor important for stress resistance. Therefore, these K1 and EJ97 resistant bacteria should have reduced fitness in an infected host. Other bacteriocins, like EF478, which is a serine protease that hydrolyses peptidoglycan, and BLIS, are active against VSE, VRE, and even certain pan-drug-resistant enterococci [86,117].

An interesting approach also consists of the use of bacteriocins in combination with antibiotics, especially if the two drugs are synergistic. Many bacteriocins are being studied, such as CSpK14, which allows the sensitization of VRE and VRSA to β-lactams [87] and AS-48 active against VRE (phenotypes VanA, B, and C) in combination with vancomycin, gentamicin, or amoxicillin/clavulanate [88]. AS-48 has the added advantage that it does not show cross-resistance with the tested antibiotics.

Bacteriocins offer several advantages in the treatment of resistant pathogens. They are generally stable over a wide pH range, withstand high temperatures (60 °C for 1 h for EF478), and are not toxic for humans. Their target is often different from those of traditional antibiotics. Their MoA can be either broad or narrow, which can be an advantage in treating an infection without disrupting the patient’s microbiota. In addition, their protein nature allows easier modifications of the molecules than for antibiotics [19]. 

A disadvantage of using bacteriocin producing strains is the proximity of the producing and target strains which increase the risk of transfers of bacteriocins and associated resistances. This is particularly the case with the bacteriocins Bac32 and Bac43. The genes coding for these bacteriocins are on transferable plasmids allowing their biosynthesis and associated immunity that can be transferred by conjugation to other bacteria. Another problem linked to this conjugation is that these plasmids are generally co-transferred with a plasmid conferring gentamicin resistance for Bac32 and vancomycin resistance for Bac43 [118,119]. The recipient bacteria therefore acquired both bacteriocin and antibiotic resistances. Another disadvantage of bacteriocins is that, as antimicrobial peptides in general, their protein nature should make them susceptible to proteases inside an infected host. Some bacteriocins are highly stable to proteases, like pseudomycoicidin, a lantibiotic, which is resistant to trypsin [120] or lasso peptides in general that are totally or partially resistant to enzymatic degradation [121,122].

#### 2.2.2. Bacteriophages

Bacteriophages (or phages) are viruses that target one or more species of bacteria. The infection of a bacterium by a phage leads to its lysis, so they can be used as a treatment for certain pathogens. Their cycle can be lysogenic or lytic, but only lytic phages are used clinically [95]. The first therapies took place in the 1920s, but several limitations as well as the discovery of antibiotics, hampered the development of these therapies in Western countries [94]. These therapies continued to be developed in the Eastern Bloc during the cold war and are still in use today in these countries. Due to the emergence of antibiotic resistant bacteria these phago-therapies are currently experiencing a renaissance with several clinical trials underway around the world [123,124,125].

A strategy to prevent the spread of resistance to vancomycin is the use of a phage (vrep-5) to treat livestock compost to eliminate VRE. Since farm animals were formally treated with avoparcin, a vancomycin analogue, strains of VRE were found in the compost which, once scattered, contaminated neighboring soils and surrounding areas [126].

The application of phages seems to also be a promising strategy to prevent or fight infections due to vancomycin resistant pathogens. Phage treatments can be divided into two approaches. The first is based on the endolysins (or lysins) of the phages of interest. During the infection process of the bacteriophage, the lysins are exported from the bacterial cell. Their substrate is the peptidoglycan and its degradation causes lysis of the bacterium and release of the virions. Several recombinant lysins have been tested against VRE. For example, the lysins, Lys168 and Lys170, have an activity on most of the tested strains of *E. faecalis* and several strains of *E. faecium*, including vancomycin resistant strains [89]. Another lysin, ORF28, derived from phage ϕEf11, has muramidase, glucosaminidase, and endopeptidase activity and acts on vancomycin resistant *E. faecalis* but has limited effects on *E. faecium* [90]. Other lysins are under study, such as LysSAP26 from phage SAP-26, which has activity on VRE [91]. These lysins have many advantages over conventional antibiotics. First, their target is the peptidoglycan of bacteria, an essential component of bacterial cells, and they act quickly, reducing the risk of developing resistance. In addition, their target (peptidoglycan) is specific for bacteria, so they are not toxic to eukaryotes. As their spectrum of action is limited to a few species, they are also more specific than antibiotics [90]. They can be produced via heterologous expression in *E. coli*, which allows increased production [89]. However, resistance to phage endolysins might be due to the presence of prophages in the targeted bacteria encoding the lysins as reported for phage ϕEf11 [92]. Lysins from temperate phages may therefore be less useful.

Lysins could also be used in combination with bacteriocins, as the Tgl lysin of the MSA6 phage active against VISA, which, associated with nisin, decreases the survivability of *S. aureus* cells [93].

The second option is to treat infections with whole phages. Many phages are isolated and characterized, such as LM99, active against VRE, ΦEF24C, active against vancomycin resistant *E. faecalis*, or CoNShP-3, active against coagulase negative Staphylococci [127,128,129]. The lysins of these phages have both endopeptidase and amidase activity and act on some VRSA and MRSA even when organized in biofilms [129]. Some phages have been tested on mice with VRE bacteremia such as ENB6, which allowed 100% survival of infected mice and rescued 50% of moribund animals [94]. The pair of phages, EFDG1/EFLK1, has been particularly studied. EFDG1 is active against VRE and their biofilms [95]. Resistance to EFDG1 phage was observed after treatment which led to the discovery of phage EFLK1, which showed an activity on strains resistant to EFDG1 [96]. These two phages were given as a cocktail to mice with peritonitis caused by VRE and a single dose resulted in 100% survival if the treatment was given within the first hour of infection, and 60% survival if it was given 6 h later [96]. 

Another treatment strategy consists of a combination of one or more phages with antibiotics. The addition of ampicillin to the phage cocktail reduced bacterial load in mouse organs greater than phage cocktail or ampicillin alone [96]. The combination of EFLK1 with vancomycin allows a synergy of treatment in planktonic VRE, and is more effective than each separate antimicrobial on biofilms. This also greatly reduced the required concentrations of vancomycin [98]. 

There are drawbacks to the use of phages. The narrow spectrum of action of phages requires isolating the pathogen in order to find an appropriate phage highly lytic for the isolate. This increases patient management time significantly [130]. It is also necessary to sequence the phage genome in order to verify the absence of genes for toxins or resistance to antibiotics. Horizontal gene transfer of antimicrobial resistance genes by phage transduction is also possible and could disseminate these genes among bacterial populations [131]. In addition, phage resistance can quickly appear in target bacteria [132,133]. The site of infection is also important, because phages should have access to the pathogen to be efficient. It has been shown that some phages and their bacterial target could coexist in the gut of mice [134]. Conversely, intravenous administration of phages seemed to result in better outcomes for patients in the case of sepsis caused by *S. aureus* [135].

Some phages can trigger an immune response in the host, which could still allow better elimination of the infection since the non-specific immune response will eliminate both phages and pathogens [94,96,99,136]. The use of phage cocktails or combinations with antibiotics increase selection pressures and therefore decrease the probability for appearance of bacteria resistant to any component of the treatment [96,97]. Phages are also considered as self-propagating antimicrobials [129], since their abundance is correlated to the target population and therefore a single injection of phage(s) should be enough to treat patients [95]. Another advantage of phages and their lysins is the ease to create variants by directed or non-directed mutagenesis (UV passages, chemical mutagens, etc.). A new platform technology has been developed to create on demand phages with custom genome, allowing the creation of libraries of phages [137]. Libraries of variants can then be screened for more efficient phages or lysins [97]. Many phages can also act on biofilms, which are often resistant to antibiotics [95]. The use of personalized phage cocktails would also limit the appearance of resistance before complete curing of the infection since all phages will be active on the corresponding pathogen. Although currently phages for Gram-negative pathogens seems easier to isolate, the increased interest in phagotherapy should also accelerate the isolation of lytic phages infecting Gram-positive pathogens [132].

#### 2.2.3. Nanoparticles

Numerous studies to evaluate the effectiveness of nanoparticle-based treatments are currently conducted on various pathogens. These treatments are promising due to the wide variety of nanoparticles (NPs) with different MoAs available (Figure 1, Table 1) [100]. Approaches based on NPs have been extensively reviewed previously [100] and therefore we will focus here on those effective on VRE or VRSA.

Several types of NPs exist. Metallic NPs can be composed of various metals, such as zinc, silver, gold, titanium, magnesium, or copper and others are composed of polymers [102,138]. There are also NPs loaded with traditional antibiotics.

Concerning metal NPs, they can have various MoAs. NPs containing silver elicit the generation of reactive oxygen species (ROS) inside bacteria leading to cell death. In addition, silver ions (Ag+) can create pores in bacterial membranes, and inhibit DNA replication by binding to DNA [100,139,140]. In addition to ROS, some NPs can generate reactive nitrogen species which have many MoAs on the metabolism of bacteria [100]. Some zinc oxide (ZnO) NPs are active on VRE and *S. aureus* [141]. Other NPs act on VRE but some of them are only active at concentrations also toxic for eukaryotic cells [142].

Different polymer NPs have very diverse MoAs. For example, chitosan NPs can cause membrane disruption in *P. acnes* [101]. They can also bind to the DNA of the bacteria to inhibit transcription or act as a metal chelator [100,101]. Another NP, named DA95B5, is a non-bactericidal “block copolymer nanoparticle” capable of eradicating biofilms of Gram-positive bacteria, including VRE. It binds to the surface of the bacterial cells thereby preventing interaction with the matrix without causing damage to the bacteria. Such a treatment would be particularly interesting for skin infections [102].

NPs can also be used in combination with antibiotics or AMPs. These drugs can be given separately or loaded onto the NP. For example, zinc NPs used in combination with vancomycin delivered promising results on enterococci [143]. NPs loaded with silver ions and an antimicrobial peptide copolymer have a multimodal effect on VRE including membrane damage and ROS production resulting in killing of the bacteria in vitro and in a mouse model. These NPs were found to be of low toxicity for eukaryotic cells and no resistance was observed in the bacteria studied after 21 passages in the presence of the NPs [49]. Gold NPs associated with different antibiotics including vancomycin were active on VRE in vitro without being toxic for macrophages [144,145]. One of these vancomycin-loaded gold NPs have been shown to be more active on VRE than vancomycin alone and to have activity on *S. aureus* inside macrophages [144]. Furthermore, NPs made of self-assembled amphiphilic peptides have an antibacterial effect on *E. faecalis*, including VRE [103]. Furthermore, vancomycin-conjugated chitosan NPs seem to have an effect on VRSA [100,146]. 

Technical progress and the urgent need of alternatives to antibiotics has intensified the research on NPs as therapeutics against MDR pathogens. Many efforts are currently being undertaken to synthesize NPs in a single environmentally friendly step to facilitate their production [138,141]. The wide spectrum of NPs already synthesized allows having many different MoAs at our disposal, and NPs can be used to encapsulate antibiotics in order to improve their delivery [147]. Combinations with antibacterials increase the versatility of NPs. Although few studies reported toxic effects of NPs for eukaryotic organisms, so far this technology has not been approved for use in human medicine.

## 3. Conclusions

The awareness of the generalization of the antibiotic resistance acquisition by bacteria has stimulated the development of many antimicrobials and very diverse approaches, including in the fight against pathogens resistant to vancomycin.

In general, antibiotics constitute a short-term approach to counter this resistance because their development until introduction into the market is long and resistance appearance is generally fast. The solution of the combination of antibiotics makes it possible to diversify the MoA and thus limit the appearance of resistance, but this solution is far from being perfect due to the broad spectrum of action of such a treatment, leaving the patient with altered microbiota and possible increased of the risk of subsequent infections [148]. Some antibiotic molecules still have a chance to replace vancomycin as the last-line antibiotic, such as the vancomycin-derived molecule from Okano et al. which, due to its multiple synergistic MoA, can treat infections caused by pathogens resistant to vancomycin while preventing the development of resistance [61]. Without coordination between players in the antimicrobial sector, these types of molecules will however end up being overtaken by bacteria that have accumulated resistance to antibiotics sharing an MoA with these “super antibiotics”.

The development of research around unconventional antimicrobials has therefore logically increased with the aim of finding a viable solution to the problem of MDR bacteria. While bacteriocins may have the same problem as antibiotics if their use increases, some antimicrobials could play an important role in fighting antibiotic-resistant bacteria. Antimicrobial peptides and NPs, for example, have the advantage of being able to act physically on bacteria, without the need for a specific bacterial target, reducing the risk of resistance appearance. However, once in the host, a toxicity problem is possible [112,142]. Conversely, bacteriophages target only bacteria and are harmless to the host even if immune reactions have already been observed [94]. This technology is perhaps the most promising of those presented here, because one injection is enough to treat the pathogens, and even in the case of a resistance, a phage effective against the resistant bacteria will be able to emerge quickly. The use of phage cocktails also helps reduce the risk of resistance development. The problem with this technology is that identification of the pathogen and its sensitivity to a given phage is required, delaying the start of patients’ treatment, and the treatment site conditions the efficacy of phages.

Some approaches reduce the emergence or spread of vancomycin resistant strains, as shown by the vancomycin antagonist peptide and phage vrep-5 [75,80,126]. Although these potential treatments come too late to prevent vancomycin resistance, the approach is interesting because it can still prevent the spread of it and it should be developed in conjunction with new antimicrobials. The development of anti-virulence treatments could also constitute a lasting alternative to current treatments because they would greatly decrease the selection pressure. Indeed, such treatment would not kill the pathogen but would prevent its virulence, reducing the selective pressure.

There are currently alternatives to vancomycin (linezolid, oritavancin, etc.), but the risk of resistance is to be expected. Therefore, the development of new antimicrobials and treatment strategies is important to pursue.

## Figures and Tables

**Table 1:** Sum up of the antimicrobials presented in this review. Bold text means that all the antimicrobials of the same type are concerned.

Antimicrobial	Utilization	Target	Mechanism of Action	Pros	Cons	References
Available antibiotics						
linezolid	clinical use	23S ribosomal RNA	protein synthesis inhibition	**- well known** **- industrial production**	**resistance**	[20,21,22,23,24,25,26,28,29,30,31,32,36]
daptomycin	clinical use	calcium ions	membrane disruption			[20,22,29,35,37,38,39,40,41,42,43]
tigecycline	clinical use	30S ribosomal subunit	protein synthesis inhibition			[20,44,45,46,47]
tedizolid	clinical use	23S ribosomal RNA	protein synthesis inhibition	active against linezolid resistant bacteria (*cfr* gene)		[33,48]
Modified antibiotics						
vancomycin dimers	in vitro (in vivo on *S. pneumoniae*) [49]	Penicillin binding protein 2	cell wall synthesis inhibition	new mechanism of action	resistance is still possible	[50,51,52,53]
teicoplanin derivatives	in vitro	lipid II		active on VanA phenotype		[54]
dalbavancin	clinical use	lipid II			- increasing MIC - resistance of VanA phenotype	[55,56,57,58]
oritavancin	clinical use	lipid II—membrane	- cell wall synthesis inhibition (transglycosylation and transpeptidation) - membrane disruption	3 different mechanisms of action	resistance in vitro	[55,56,58,59,60]
vancomycin derivative	in vitro	lipid II (D-ala-D-ala and D-ala-D-lac)—membrane	- cell wall synthesis inhibition - membrane disruption	3 different mechanisms of action		[61]
Combination of antibiotics						
various types and classes	clinical use	DNA/RNA/protein metabolism—membrane	multiple	- multiple mechanisms of action - overcome resistance	wide spectrum	[22,43,62,63,64,65,66,67,68,69,70,71,72,73,74]
Antimicrobial peptides						
anti-vancomycin peptide	in vivo	vancomycin	vancomycin concentration decreasing	prevent the emergence of resistance		[75]
SLAY-P1	in vivo	VanRS	vancomycin resistance inhibition	- overcome resistance - stable in human serum	resistance is still possible	[76]
cationic peptides	in vivo	membrane	membrane disruption	- physical interactions **- easy engineering**	- stability in host - toxicity **- production cost** **- resistance**	[76]
peptides + antibiotics combination	in vivo	DNA/RNA/protein metabolism—membrane	multiple	- multiple mechanisms of action - overcome resistance - activity on biofilm	resistance is still possible	[76,77,78,79]
Bacteriocins						
bacterial transplantation	in vivo	intestinal VRE	VRE elimination	resistant pathogen elimination before infection	**- resistance is still possible** **- sensitive to proteases**	[80]
mersacidin	in vivo	lipid II	cell wall synthesis inhibition			[81,82]
lacticin 3147	in vivo	lipid II	membrane disruption	active against multiple pathogens of interest		[81,83]
pumilicin 4	(clinical use = *B. pumilus* probiotic)	unknown	membrane destabilization	heat resistant		[84]
K1 and EJ97	in vitro	RseP	membrane depolarization	attenuates bacteria’s pathogenicity	narrow spectrum	[19,85]
EF478	in vitro	peptidoglycan	cell wall disruption	active against MDRE		[86]
bacteriocins + antibiotics combination	in vitro	DNA/RNA/protein metabolism—membrane	multiple	- multiple mechanisms of action - overcome resistance - activity on biofilm **- used in food for more than 50 years** **- generally stable** **- easy engineering** **- non toxic**		[87,88]
Lysins						
	in vivo	cell wall	peptidoglycan lysis	- act quickly = less resistance - heterologous production - easy engineering	resistance	[89,90,91,92]
combination lysin bacteriocin	in vitro	cell wall	peptidoglycan lysis			[93]
Bacteriophages						
ENB6	in vivo	cell metabolism				[94]
EFDG1/EFLK1	in vivo	cell metabolism		- can act on biofilm - overcome resistance		[95,96,97]
combination phages antibiotics	in vivo	- cell metabolism - DNA/RNA/protein metabolism—membrane	**hijack of the cell metabolism + other depending on the antibiotic**	- overcome resistance - reduce antibiotics concentrations	wide spectrum	[96,98]
all bacteriophages	clinical use	cell metabolism	**hijack of the cell metabolism**	**- “smart therapy”** **- only one administration**	**- host immune response** **- possible toxicity with toxins** **- narrow spectrum = isolation needed**	
Nanoparticles						
silver nanoparticles	in vivo	- membrane - cell metabolism	- membrane disruption - inhibition of DNA replication - ROS production	multiple mechanisms of action	**possible toxicity**	[99]
chitosan nanoparticles	in vitro	- membrane - cell metabolism	- membrane disruption (*P. acnes*) - transcription inhibition - metal chelation	multiple mechanisms of action		[100,101]
AgNPs@PCL-b-AMPs	in vivo	- membrane - cell metabolism	- membrane disruption - ROS production	- no toxicity to eukaryotic cells - no resistance in vitro		[49]
DA95B5	in vivo	cell surface	biofilm inhibition	- no toxicity - limitation of biofilm	need another treatment to kill bacteria	[102]
polypeptide-based nanoparticles	in vitro	membrane	membrane disruption	- no nanoparticles resistance observed - low toxicity		[103]

## Data Availability

Data sharing not applicable.
